# Multifunctional twelve port frequency agile diversity antenna for indoor wireless applications

**DOI:** 10.1038/s41598-023-34945-8

**Published:** 2023-05-17

**Authors:** Deepa Thangarasu, Sandeep Kumar Palaniswamy, Rama Rao Thipparaju, Mohammed S. Alzaidi, Sachin Kumar, Dalia H. Elkamchouchi

**Affiliations:** 1 Department of Electronics and Communication Engineering, SRM Institute of Science and Technology, Kattankulathur, Tamil Nadu 603203 India; 2 Department of Electrical Engineering, College of Engineering, Taif University, P.O. Box 11099, Taif, 21944 Saudi Arabia; 3 Department of Information Technology, College of Computer and Information Sciences, Princess Nourah Bint Abdulrahman University, P.O. Box 84428, Riyadh, 11671 Saudi Arabia

**Keywords:** Engineering, Physics

## Abstract

The recent resurgence of new-generation reconfigurable technologies delivers a plethora of various applications in all public, private and enterprise solutions over the globe. In this paper, a frequency reconfigurable polarization and pattern diverse Multiple-Input-Multiple-Output (MIMO) antenna is presented for indoor scenarios. The MIMO antenna is comprised of twelve radiating elements, and polarization and pattern diversity is obtained by arranging them in three different planes: Horizontal Plane (HP), Vertical Plane-I (VP-I), and Vertical Plane-II (VP-II). The proposed antenna operates in mode I (wideband) and mode II (multiband), by combining two different radiators using PIN diodes. The antenna dynamically switches between Mode I (wideband) and mode II (multiband). Mode, I cover the ultra-wideband (UWB) range from 2.3 to 12 GHz, while mode II covers GSM (1.85–1.9 GHz), Wi-Fi and LTE-7 (2.419–2.96 GHz), 5G (3.15–3.28 GHz and 3.45–3.57 GHz), public safety WLAN (4.817–4.94 GHz), and WLAN (5.11–5.4 GHz) frequency bands. The peak gain and efficiency of the MIMO antenna are 5.2 dBi and 80%, respectively.

## Introduction

Due to rapid advancements in the wireless world, to resolve connectivity issues, high data rates, power constraints, miniaturization, and multi-serviceability. Antenna modules have to support transmission and reception simultaneously to provide uninterrupted service to the user. Especially indoor scenarios such as shopping malls, airports, universities, industries, schools, hospitals etc., encounters more connectivity issues^1–3^ due to small-scale fading. However, these significant challenges are due to multipath propagation, which reduces the signal-to-noise ratio and affects link reliability due to polarization mismatch. The fading effect can be mitigated by introducing spatial diversity at the transceivers. Therefore, Multiple-Input-Multiple-Output (MIMO) diversity antennas are used in wireless transceivers to improve communication reliability^4–8^. From the literature of similar research works, MIMO antennas are categorized as wideband^9–14^, multiband^15–17^, and integrated^18–20^, which is a combination of wideband and multiband.

Wideband MIMO antennas are widely used in modern wireless systems due to their multiple advantages, including high data rate transmission and low power consumption. In^9^, a quad-element MIMO antenna that covered the 5G band and the C-band in two different states using an LC tank circuit was reported. In^10^, a slot-based quad-element MIMO antenna was developed for cognitive radio applications. However, the antenna lacked polarization diversity. In^11^, a compact MIMO antenna was designed to cover the ultra-wideband (UWB) range with a notched band at 5.5 GHz. In^12^, an antenna array covering the UWB was reported, and a narrow slot was introduced to achieve high isolation between the unit cells. In^13^, a four-element UWB MIMO antenna with high isolation between antenna elements was proposed. In^14^, an eight-port 3-D UWB MIMO antenna with polarization was presented. In^15^, an eight-element MIMO/diversity antenna was reported with WLAN rejection, which covered the 3G, 4G, and 5G frequency bands. In^16^, a two-element multiband antenna with decoupling structures was developed for the smartphone. In^17^, a four-element MIMO antenna with a meandering, and split-ring resonator was reported with high inter-element isolation. However, the majority of above reported MIMO antennas had complicated geometry, and large size, and employed complex decoupling structures.

Recently, Integrated MIMO Antennas (IMA) have received a lot of attention due to their high-speed data transmission and multi-serviceability. These antennas offer both wideband and narrowband characteristics and are useful for IoT modules. However, only a few IMA designs, integrating multiple bands into a single entity, are reported in the open literature. In^18^, a twelve-port MIMO antenna was reported with five pairs of single and dual band antenna elements and two UWB antenna elements. In^19^, a MIMO antenna with twelve elements was reported for UWB, GSM, and Bluetooth standards. In^20^, an eight-element frequency reconfigurable polarization diversity IMA was designed for vehicular communication applications. However, the antenna designs reported in^18–20^ had a large size, limited functionality, and limited diversity. The present-day modules must support a wide range of wireless communication standards, integrating a large number of resonating elements with the smallest possible antenna size and minimum inter-element interference. Therefore, reconfigurable antennas could be suitable for indoor scenarios as they are adaptable to user demands. The reconfigurable IMA benefits in the following ways: (i) integrating more radiators in a small space, (ii) enabling multi-serviceability by changing the frequency, pattern, and polarization^21,22^, (iii) improving dynamic spectrum accessibility based on user demand^23–25^, and (iv) offering filtering characteristics within the MIMO antenna to avoid interferences. Also, metasurface-based concepts were reported in^26–28^ to improve the antenna performance.

In this research, a twelve-port frequency reconfigurable MIMO antenna is presented for high scattering environments to offer 360° coverage. The proposed antenna is comprised of two radiators: Radiator-I, a modified U-shaped patch that covers the entire UWB spectrum, and radiator-II, which is made up of meandering lines (L-shaped, F-shaped, and open-ended) to support multiple wireless standards, including 2G, 4G, 5G, Wi-Fi, Public safety WLAN and WLAN. Also, the twelve antenna elements are oriented in a 3-D fashion to minimize the probability to occur polarization mismatch and coverage issues in ultra-dense environments.

### Antenna design

**Figure 1:**
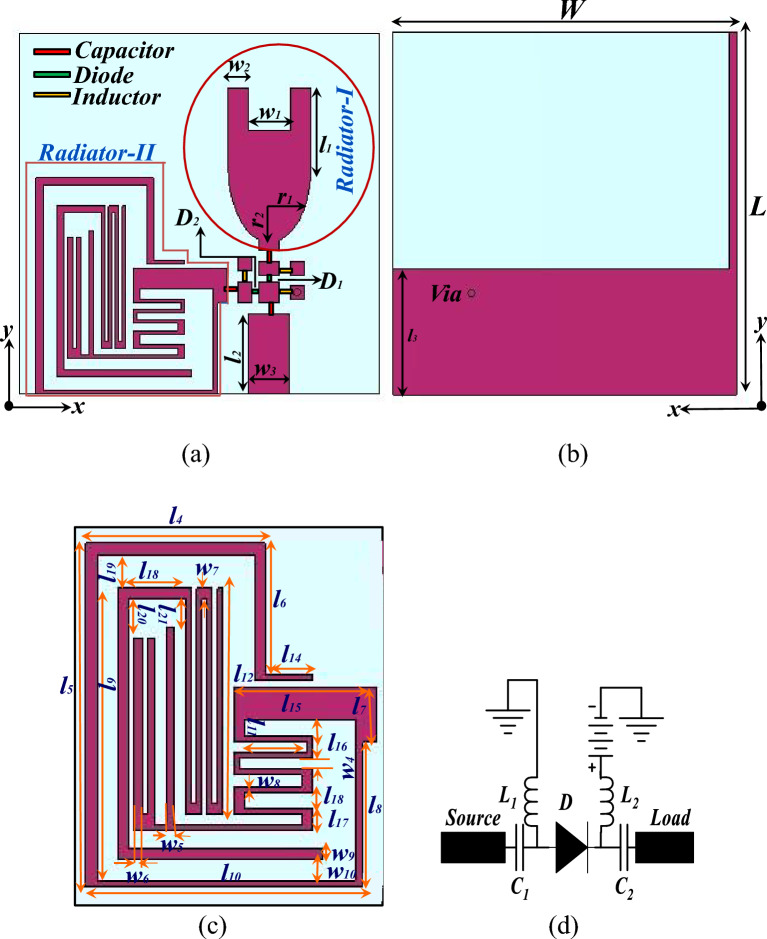
Schematic of the antenna element (**a**) top view, (**b**) bottom view, (**c**) enlarged view of the multiband radiator, (**d**) equivalent circuit of the PIN diode.

The schematic of the antenna element is shown in Fig. 1. The proposed antenna is printed on the FR-4 substrate with relative permittivity of 4.4 and a thickness of 1.6 mm. The antenna consists of two radiators that operate over the UWB and six narrow bands to support multiple wireless applications. Switching between the two radiators is achieved using the surface mountable PIN diodes (SMP1320-079) denoted as D1 and D2 in Fig. 1a. The PIN diodes are chosen due to their fast switching, low resistance of 0.9 Ω, and low inductance of 0.4 nH. A biasing circuit is also designed, with blocking capacitors C1 and C2 of 20 pF and RF chokes L1 and L2 of 33 nH. The ground plane of the antenna element is depicted in Fig. 1b, and the dimensions of the multiband radiator are displayed in Fig. 1c. The antenna simulations are performed in the CST Microwave Studio^®^ software, and the antenna dimensions (in mm) are as follows: L = 26, W = 26, r1 = 3, r2 = 5.5, l1 = 4.9, l2 = 5.75, l3 = 13, l4 = 8.5, l5 = 15.5, l6 = 6, l7 = 2.5, l8 = 6.55, l9 = 12, l10 = 13, l11 = 3.25, l12 = 10.2, l13 = 8.5, l14 = 2.25, l15 = 6.75, l16 = 1, l17 = 1, l18 = 3.5, l19 = 1.8, l20 = 1.77, l21 = 1.37, w1 = 3, w2 = 1.5, w3 = 3, w4 = 0.35, w5 = 0.25, w6 = 0.55, w7 = 0.5, w8 = 0.25, w9 = 0.5. The equivalent circuit of the PIN diode is shown in Fig. 1d. The design equation for the proposed antenna is represented as,

1 $$f_{l} = \frac{c}{{(m + l_{1} \times p) \times k}}$$

here ${\text{k}} = \sqrt[4]{{\varepsilon_{reff} }},\;l_{1} = 1.414\sqrt {r_{2}^{2} } + r_{1}^{2};$ *m* = (*w* + *l*) × *p*; where *p* denotes the distance between the radiator and the ground plane, ${r}_{2}$ indicates the semi-major axis, ${r}_{1}$ indicates the semi-minor axis, and *W* and *l* denote the length and width of the proposed antenna, respectively.

### Radiator I

**Figure 2:**
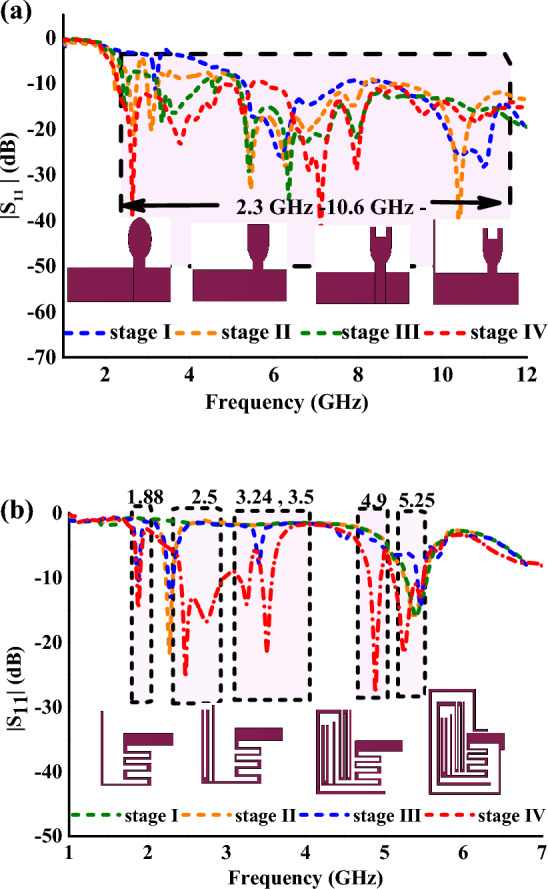
(**a**) Evolution of the radiator-I and reflection coefficients during the development. (**b**) Evolution of the radiator-II and reflection coefficients during the development stages.

The radiator-I demonstrates the UWB (mode I) operation of the proposed antenna element. Figure 2 depicts the evolution of the radiator-I and reflection coefficients during the development stages. The evolution begins with an elliptical-shaped radiator (stage I), which is fed by a microstrip line of 50 Ω, and a partial ground plane. In stage II, a rectangular patch is loaded on top of the elliptical radiator to achieve a wide impedance bandwidth. In stage III, a square slot is etched out from the top edge of the radiator to increase the current length, thereby covering the lower region frequencies and thus the UWB frequency range. Finally, in stage IV, a stub is added to the ground plane of the monopole antenna to improve impedance matching, and a diode D1 is integrated at the feed line with a biasing circuit, as shown in the Fig. 1a. The proposed modified U-shaped radiator resonates over the UWB range of 2.3 to 12 GHz.

### Radiator II

The radiator-II demonstrates the multiband (mode II) operation of the proposed antenna element. The evolution of the radiator-II and reflection coefficients during the development stages are depicted in the Fig. 2a,b. The development of radiator-II begins with an L-shaped Meandering Resonator (LMR) (stage I) that resonates from 5.11 to 5.48 GHz. In stage II, the LMR is modified into an F-shaped Meandering Resonator (FMR) to achieve resonance at 2.4 GHz (2.419–2.96 GHz). In stage III, a Meandered Open-Ended Resonator (MOER) is integrated with the FMR to achieve resonance in the 1.85–1.9 GHz frequency range. Furthermore, in stage IV, an Open-Ended Outer Resonator (OEOR), which resonates at 3.5 GHz, is integrated with the stage III radiator. Also, the gap between the LMR, FMR, MOER, and OEOR is optimized to obtain an additional resonance at 4.9 GHz.

**Figure 3:**
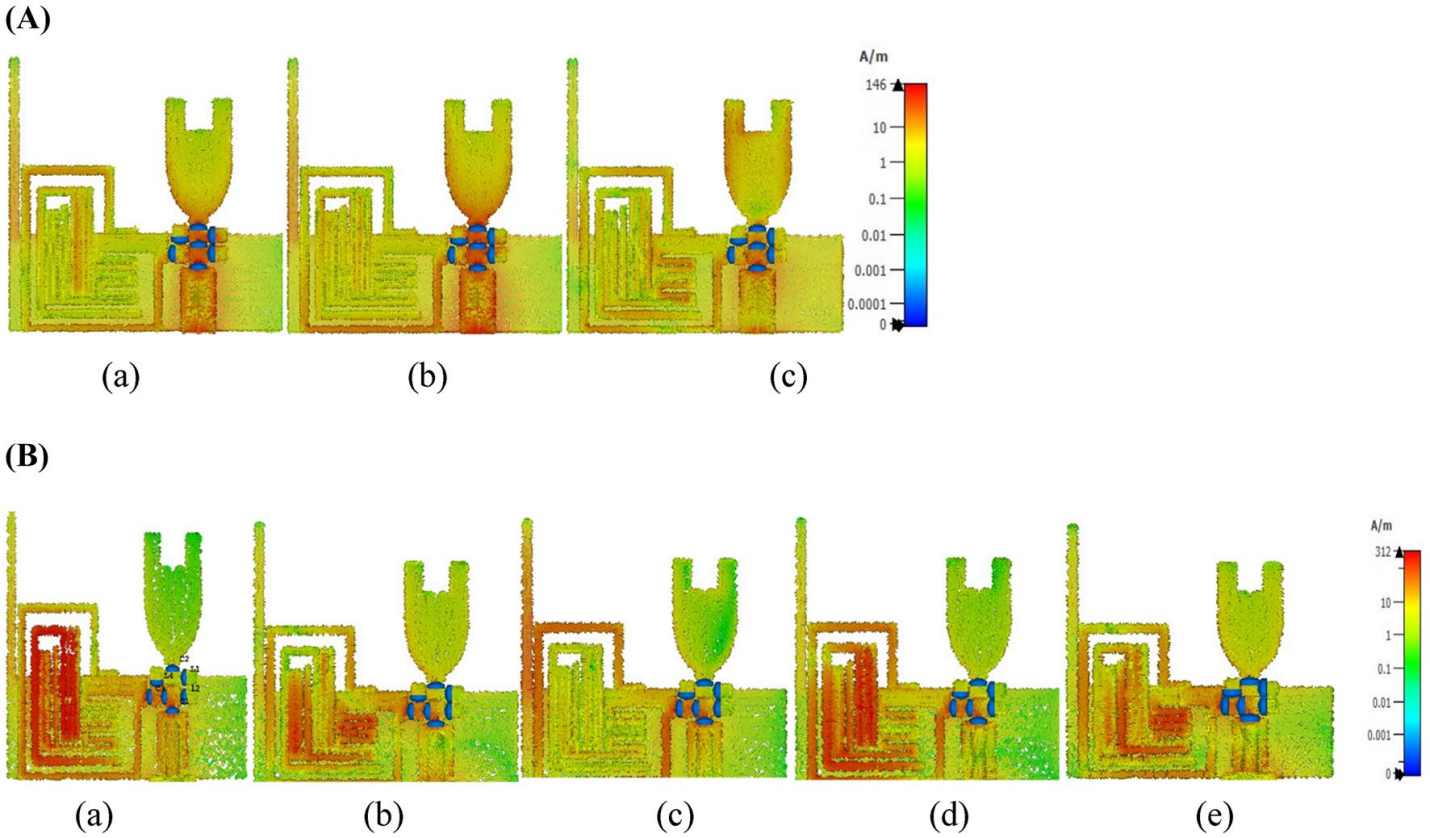
(**A**) Surface current density graphs of radiator-I at (**a**) 3 GHz, (**b**) 6 GHz, (**c**) 12 GHz. (**B**) Surface current density graphs of radiator-II at (**a**) 1.88 GHz, (**b**) 2.5 GHz, (**c**) 3.5 GHz, (**d**) 4.9 GHz, (**e**) 5.25 GHz.

The proposed multiband radiator offers hexa-band resonance, covering a wide range of wireless applications such as 2G, 4G, 5G, Wi-Fi, Public safety WLAN, and WLAN. The proposed antenna element achieves dual functionality by integrating the diode D2. When diode D1 is forward biased and diode D2 is reverse biased, the antenna will radiate over the UWB spectrum. On the other hand, when diode D2 is forward biased and diode D1 is reverse biased, the antenna switches to mode II and radiates over the six bands. The current density plots of radiator I and radiator II are presented in Fig. 3A,B respectively to understand the resonating nature of the antenna.

**Figure 4:**
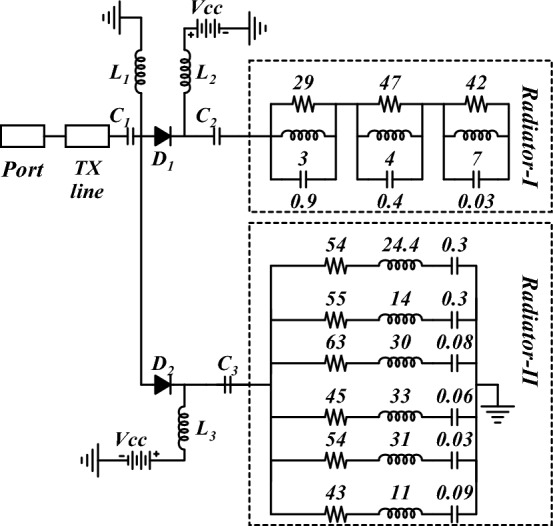
Equivalent circuit of the proposed antenna element (resistances are in Ω, inductances are in nH, and capacitances are in pF).

**Figure 5:**
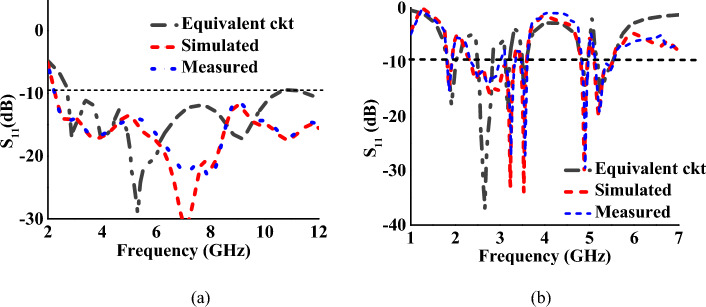
S-parameter of the equivalent circuit: (**a**) mode I, (**b**) mode II.

The equivalent circuit of the proposed antenna element is presented in Fig. 4. The lumped parameters are interpreted by predicting the type of *RLC* circuit, either parallel or series, based on the impedance characteristics of the antenna as in^29^. The three parallel resonant circuits connected in series correspond to the UWB radiator and the six series resonant circuits connected in parallel correspond to the multiband radiator. The reflection characteristics of the equivalent circuit are shown in Fig. 5 for both modes.

### Twelve port IMA

MIMO/diversity^30^ techniques are becoming increasingly important in addressing the multipath fading effects that degrade signal quality. The probability of signal fading is higher in indoor scenarios due to the existence of multiple obstructions. Hence, the polarization of the signal may change, lowering the signal quality while receiving it.

**Figure 6:**
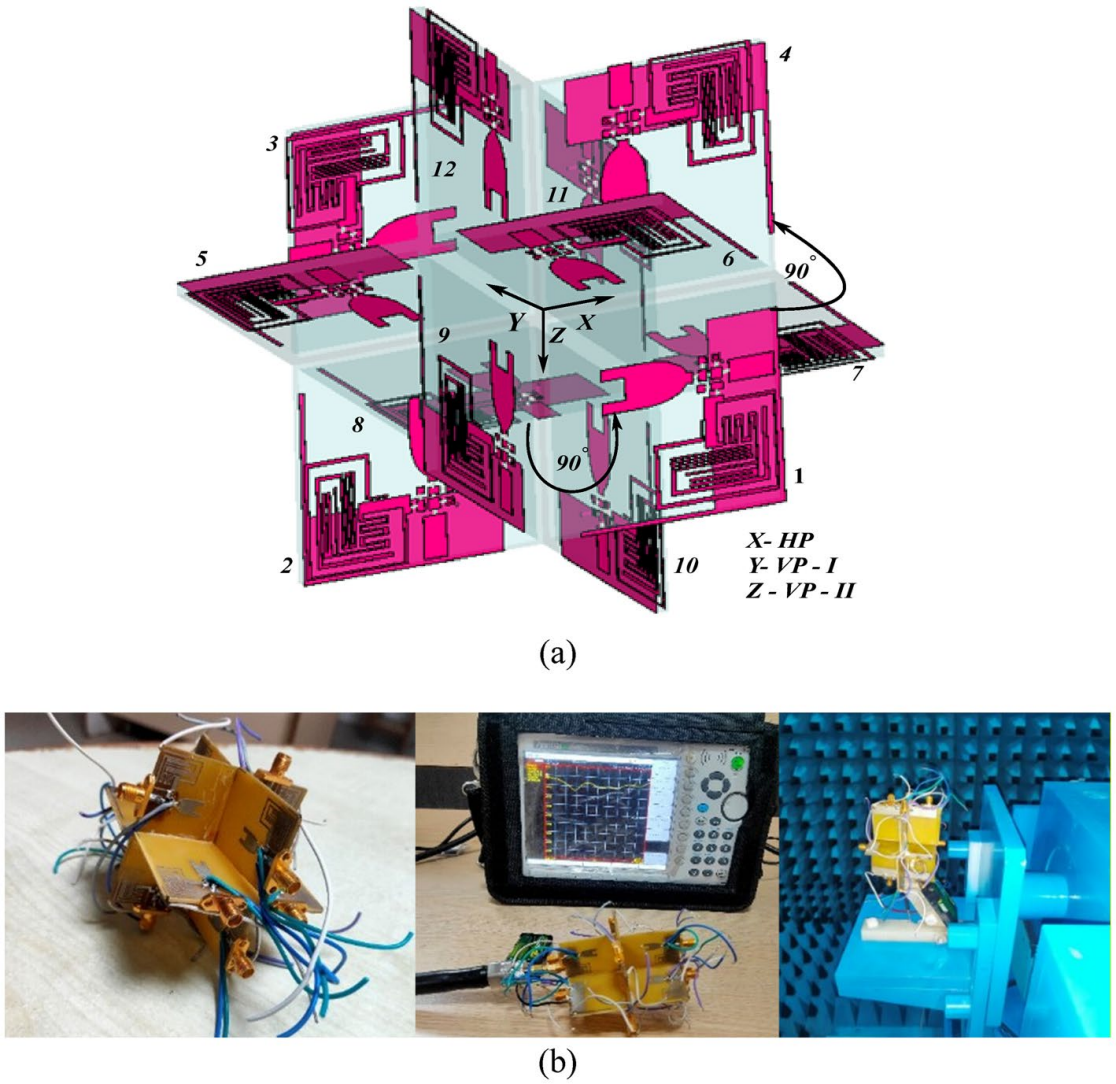
MIMO/diversity antenna (**a**) schematic, (**b**) fabricated prototype and measurement picture.

In such a situation, polarization-diverse antennas are strongly recommended to avoid polarization mismatches and increase link reliability. In this work, a twelve-port IMA is proposed with multiple polarization vectors to encounter signal losses. The schematic of the MIMO antenna is shown in Fig. 6a, and its fabricated prototype is shown in Fig. 6b. The proposed MIMO antenna consists of twelve monopole antenna elements arranged in three different planes: Horizontal Plane (HP), Vertical Plane-I (VP-I), and Vertical Plane-II (VP-II). In HP, the four resonating elements are arranged orthogonal to each other to achieve both horizontal and vertical polarization. Whereas in VP-I and VP-II, four resonating elements are interlocked in a cross-shape pattern to achieve vertical polarization. Further, the VP-I and VP-II are interlocked with the HP. However, the antenna elements in VP-I and VP-II are perpendicular to the antenna elements in HP. This particular arrangement helps in the generation of multiple polarization vectors and multiple uncorrelated beams in order to mitigate fading effects and coverage issues respectively. The overall size of the proposed IMA antenna is 62 mm × 31 mm × 31 mm.

## Results and discussion

The following subsection presents the reflection, coupling and gain characteristics of the twelve-port IMA.

### Reflection coefficients

**Figure 7:**
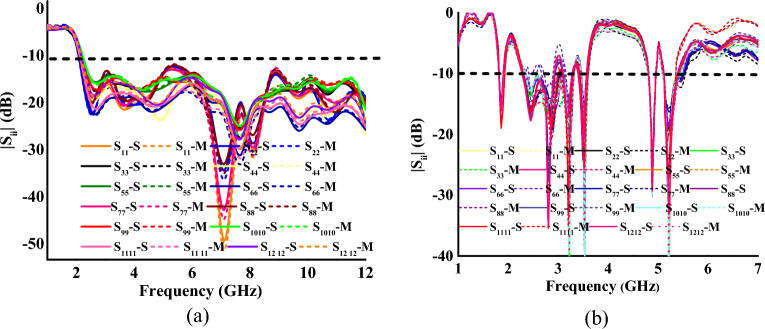
Measured and simulated reflection coefficients of the MIMO antenna (**a**) mode I, (**b**) mode II (*S* simulated, *M* measured).

Figure 7a,b display the measured and simulated reflection coefficients of the proposed MIMO antenna in mode I and mode II, respectively. In mode I, the antenna achieves an impedance bandwidth of 135% (2.3–12 GHz) over the UWB spectrum. In mode II, the antenna resonates at six bands centered at 1.88 GHz, 2.47 GHz, 2.5 GHz, 3.24 GHz, 3.5 GHz, 4.9 GHz, and 5.25 GHz, with bandwidths of 2.66%, 20.4%, 1.53%, 4.3%, 3.61%, and 7.16%, respectively.

### Mutual coupling

**Figure 8:**
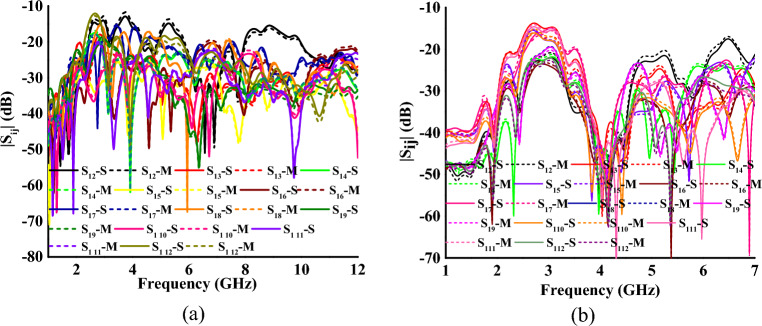
Measured and simulated coupling between the antenna elements (**a**) mode I and (**b**) mode II (*S* simulated, *M* measured).

In the proposed MIMO antenna, the four radiators are arranged orthogonally in the HP, and the remaining eight elements are placed in VP-I and VP-II. The inter-element spacing is kept as 0.16*λ*_0_, where *λ*_0_ is calculated at the lowest operating frequency. In both UWB and multiband modes, the proposed antenna achieves isolation greater than 14 dB, as shown in Fig. 8a,b.

### Antenna gain and efficiency

**Figure 9:**
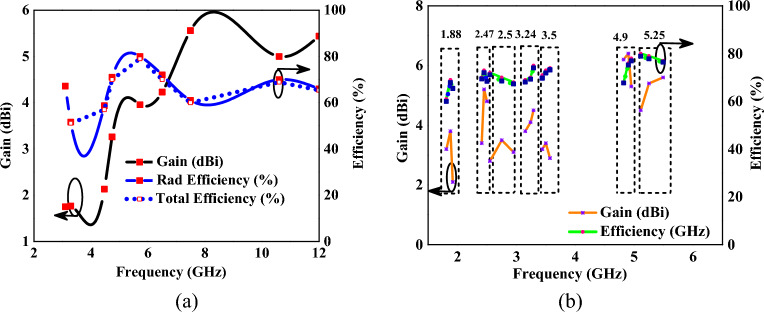
Realized gain and efficiency of MIMO antenna (**a**) mode I, (**b**) mode II.

Figure 9 depicts the gain and efficiency of the proposed antenna. In mode I, the antenna exhibits a peak gain of 5.2 dBi and an efficiency of 80%, as shown in Fig. 9a. The peak gain values in mode II are 3.8 dBi, 5.2 dBi, 3.5 dBi, 4.1 dBi, 3.41 dBi, 6.4 dBi, 5.4 dBi, and efficiency is 69%, 70%, 70%, 75%, 73%, 76%, 79% at 1.88 GHz, 2.45 GHz, 2.75 GHz, 3.24 GHz, 3.5 GHz, 4.9 GHz, 5.25 GHz, respectively, as shown in the Fig. 9b.

### Radiation characteristics

The radiation characteristics of the proposed twelve-port diversity antenna are measured in an anechoic chamber.

**Figure 10:**
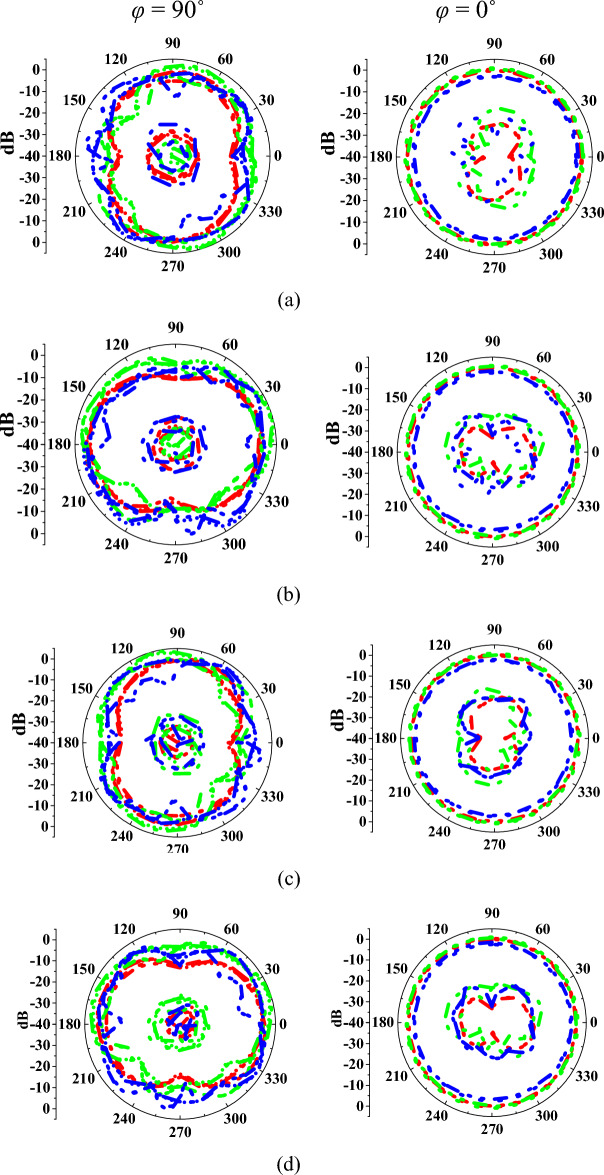

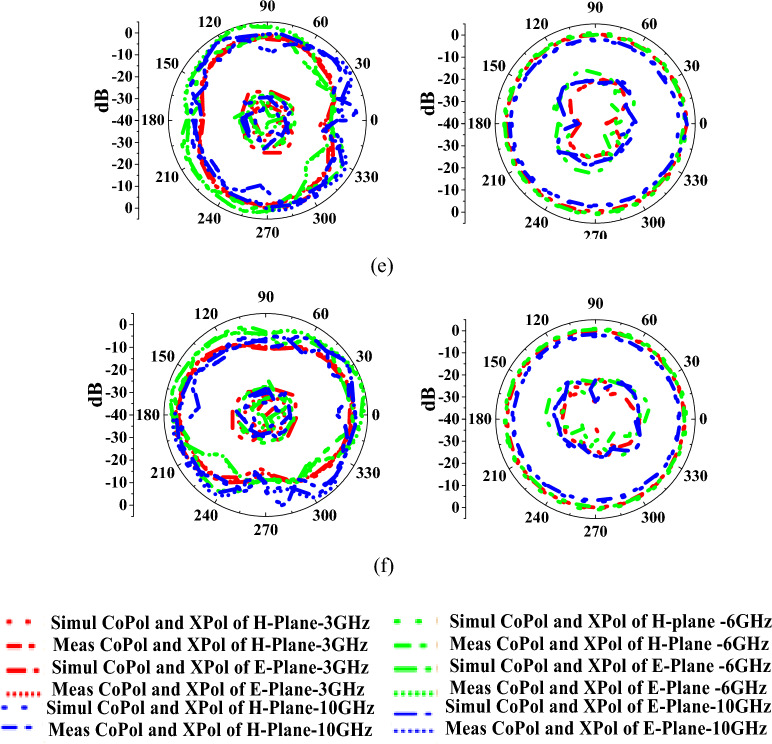
Measured and simulated radiation patterns of the antenna in mode I at (**a**) port-1, (**b**) port-2, (**c**) port-3, (**d**) port-4, (**e**) port-5, (**f**) port-12.

**Figure 11:**
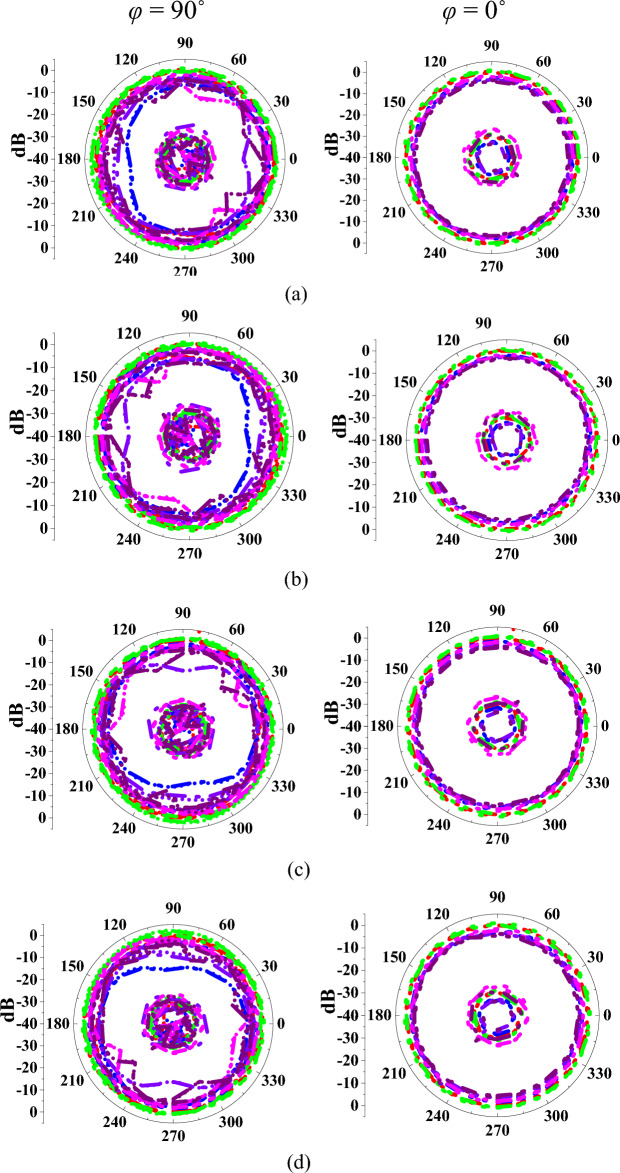

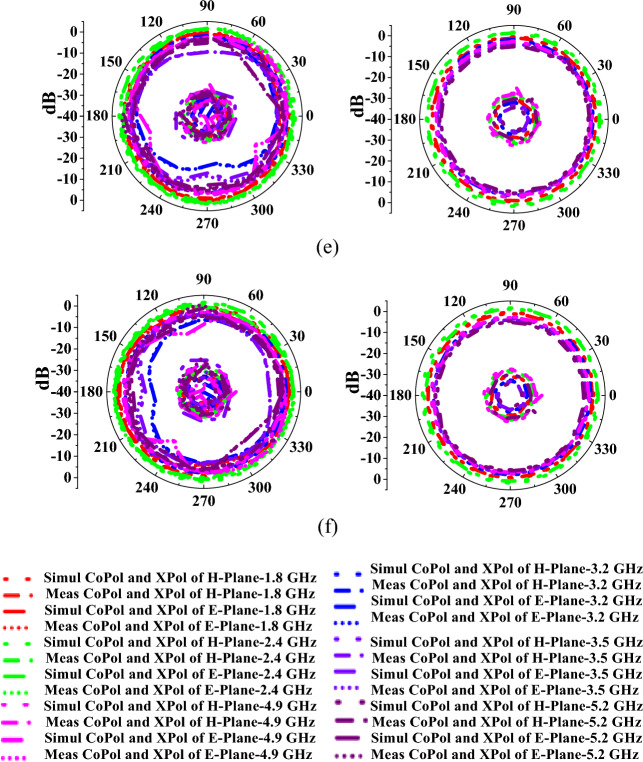
Measured and simulated radiation patterns of the antenna in mode II at (**a**) port-1, (**b**) port-2, (**c**) port-3, (**d**) port-4, (**e**) port-5, (**f**) port-12.

Figure 10 represents the antenna radiation characteristics in mode I. The radiation characteristics for port-1, port-2, port-3, port-4, port-5, and port-12 are plotted at 3 GHz, 6 GHz, and 10 GHz frequencies. Similarly, the radiation patterns for mode II are evaluated and depicted in Fig. 11. The discrepancies in radiation patterns are due to loss introduced by bias lines.

### Diversity performance of the MIMO antenna

The diversity performance of the MIMO antenna is measured using metrics such as Envelope Correlation Coefficient (ECC), Diversity Gain (DG), Mean Effective Gain (MEG), Total Active Reflection Coefficient (TARC), and Channel Capacity Loss (CCL).

### Envelope correlation coefficient (*ρ*_*e*_)

ECC^31^ is calculated using the far-field radiation characteristics of the antenna by Eq. (1).

2 $$ECC(\rho_{e} ) = \frac{{\left| {\iint {\left[ {\mathop F\limits^{ \to }_{1} \left( {\theta ,\varphi } \right).\mathop F\limits^{ \to }_{2} \left( {\theta ,\varphi } \right)} \right]d\Omega }} \right|^{2} }}{{\iint {\left| {\mathop F\limits^{ \to }_{1} \left( {\theta ,\varphi } \right)} \right|^{2} d\Omega \iint {\left| {\mathop F\limits^{ \to }_{2} \left( {\theta ,\varphi } \right)} \right|^{2} d\Omega }}}}$$

where *F* denotes the radiated field between the two antenna elements, *θ* is the angle of elevation, *φ* is the azimuthal angle, and Ω is the solid angle. The ECCs for antenna elements-2, -5, and -12 with respect to antenna element-1 are calculated for different frequencies in both modes I (3 GHz, 4 GHz, 6 GHz, 8 GHz, and 10 GHz) and II (1.88 GHz, 2.47 GHz, 3.24 GHz, 3.5 GHz, 4.9 GHz, and 5.25 GHz) and are shown in Tables 1, 2, 3, 4, 5, 6. The ECC should be 0 in the ideal case, but practically, values up to 0.5 are acceptable^29^. In all cases, the proposed MIMO/diversity antenna achieves ECC < 0.1.

**Table 1:** Diversity performance of the antenna in HP: for mode-I (between port-1 and port-2).

f (GHz)	ECC	Isolation (dB)	ADG (dB)	EDG (dB)	MEG	TARC (dB)	CCL (bits/s/Hz)
3	0.02	> 15	9.99	7.94	0.4	14	0.16
4	0.06	> 13	9.98	8.24	0.7	17	0.02
6	0.01	> 25	9.99	8.92	0.5	15	0.01
8	0.08	> 30	9.96	7.36	0.8	13	0.05
10	0.01	> 20	9.99	7.84	0.8	16	0.13

**Table 2:** Diversity performance of the antenna in VP-I: for mode-I (between port-1 and port-5).

f (GHz)	ECC	Isolation (dB)	ADG (dB)	EDG (dB)	MEG	TARC (dB)	CCL (bits/s/Hz)
3	0.01	> 30	9.99	8.94	1.3	19	0.19
4	0.08	> 40	9.99	7.24	1.7	21	0.14
6	0.06	> 25	10	7.93	1.4	27	0.07
8	0.01	> 45	9.99	7.39	0.8	18	0.16
10	0.04	> 35	10	8.84	0.6	15	0.01

**Table 3:** Diversity performance of the antenna in VP-II: for mode-I (between port-1 and port-12).

f (GHz)	ECC	Isolation (dB)	ADG (dB)	EDG (dB)	MEG	TARC (dB)	CCL (bits/s/Hz)
3	0.04	> 20	9.99	7.93	1.2	16	0.15
4	0.03	> 22	9.99	7.25	0.6	29	0.19
6	0.06	> 30	9.98	8.91	0.4	31	0.07
8	0.01	> 25	9.99	8.39	0.8	22	0.14
10	0.01	> 25	10	8.84	0.3	27	0.09

**Table 4:** Diversity performance of the antenna in HP: for mode-II (between port-1 and port-2).

f (GHz)	ECC	Isolation (dB)	ADG (dB)	EDG (dB)	MEG	TARC (dB)	CCL (bits/s/Hz)
1.88	0.09	> 35	9.95	6.81	1.9	22	0.17
2.47	0.03	> 27	9.99	2.72	1.4	23	0.13
3.24	0.08	> 30	10	5.93	1.2	25	0.18
3.5	0.05	> 31	10	3.59	0.1	34	0.06
4.9	0.02	> 38	9.99	8.90	0.7	27	0.05
5.25	0.04	> 25	9.99	7.75	0.2	24	0.22

**Table 5:** Diversity performance of the antenna in VP-I: for mode-II (between port-1 and port-5).

f (GHz)	ECC	Isolation (dB)	ADG (dB)	EDG (dB)	MEG	TARC (dB)	CCL (bits/s/Hz)
1.88	0.01	> 42	9.99	6.83	1.5	25	0.07
2.47	0.03	> 24	9.99	2.72	0.9	30	0.24
3.24	0.02	> 27	9.99	5.93	0.4	34	0.13
3.5	0.06	> 50	9.99	3.59	1.2	40	0.09
4.9	0.04	> 35	9.99	8.89	0.7	35	0.08
5.25	0.01	> 40	9.99	7.75	0.5	35	0.03

**Table 6:** Diversity performance of the antenna in VP-II: for mode-II (between port-1 and port-12).

f (GHz)	ECC	Isolation (dB)	ADG (dB)	EDG (dB)	MEG	TARC (dB)	CCL (bits/s/Hz)
1.88	0.01	> 50	10	6.83	0.3	13	0.09
2.47	0.02	> 35	9.99	2.72	0.9	18	0.22
3.24	0.04	> 30	9.99	5.93	1.5	23	0.04
3.5	0.01	> 40	9.99	3.59	1.4	29	0.03
4.9	0.02	> 35	9.99	8.9	0.7	24	0.04
5.25	0.02	> 45	9.99	7.75	0.6	23	0.01

### Apparent diversity gain, effective diversity gain and mean effective gain

Another important parameter to consider is Apparent Diversity Gain (ADG), which measures link reliability and is calculated using Eq. (3).

3 $$ADG = 10\sqrt {1 - \left| {\rho_{e} } \right|^{2} }$$

The ADG^32^ values are evaluated for mode I and mode II at different frequencies by considering ports-2, -5, and -12 with respect to port-1, and are tabulated in Tables 1, 2, ,3 ,4, 5, 6. The ADG for the proposed antenna is greater than 9.9 in all cases, indicating that the antenna can offer better link reliability. The Effective Diversity Gain (EDG) is calculated using Eqs. (3), (4), and (5).

4 $$EDG = \eta_{Total} \times ADG$$

5 $$\eta_{total} = \eta_{irad} \times \left( {1 - \sum\limits_{j = 1}^{N} {\left| {S_{ij} } \right|^{2} } } \right)$$

6 $$\eta_{irad} = \left( {1 - \sum\limits_{j = 1}^{N} {\left| {S_{ij} } \right|} } \right)$$

The EDG values for mode I and mode II at ports-2, -5, and -12 with respect to port-1 are presented in Tables 1, 2, 3, 4, 5, 6. The EDG is lower than the ADG as it takes radiation losses into account. Another important MIMO parameter is the MEG ratio, which determines the average amount of power received by the antenna in a multipath fading environment, and it can be calculated using Eq. (7).

7 $$MEG = \int_{0}^{2\pi } {\int_{0}^{\pi } {\left[ \begin{gathered} \frac{XPR}{{1 + XPR}}G_{\theta } (\theta ,\phi )P_{\theta } (\theta ,\phi ) \hfill \\ + \frac{1}{1 + XPR}G_{\theta } (\theta ,\phi )P_{\theta } (\theta ,\phi ) \hfill \\ \end{gathered} \right]} } \sin \theta d\theta d\phi$$

**Figure 12:**
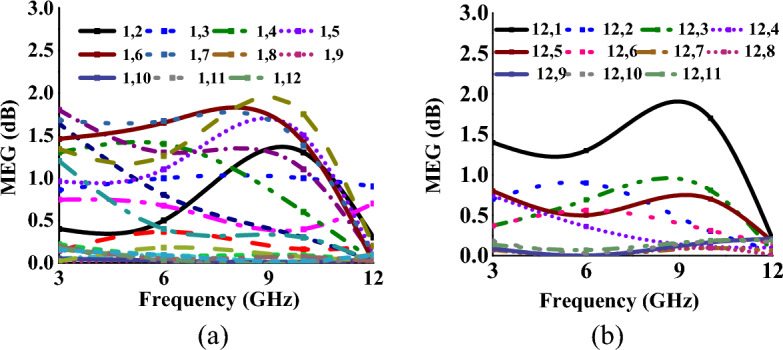
MEG characteristics at (w.r.t. other ports for mode-I: wideband) (**a**) port-1, (**b**) port-12.

**Figure 13:**
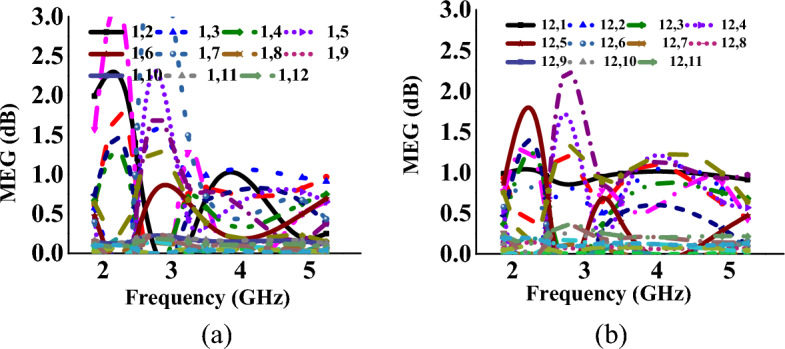
MEG characteristics at (w.r.t. other ports for mode-II: multiband) (**a**) port-1, (**b**) port-12.

The MEG values for mode I and mode II at ports-2, -5, and -12 with respect to port-1 are presented in Tables 1, 2, 3, 4, 5, 6. It is found that the diversity performance metrics are within practical limits, confirming that the proposed MIMO antenna is a good candidate for wireless indoor scenarios. Figures 12 and 13 represent the MEG characteristics of the proposed antenna for both modes, and it is found to be less than 2 dB.

### Total active reflection coefficient and channel capacity loss

**Figure 14:**
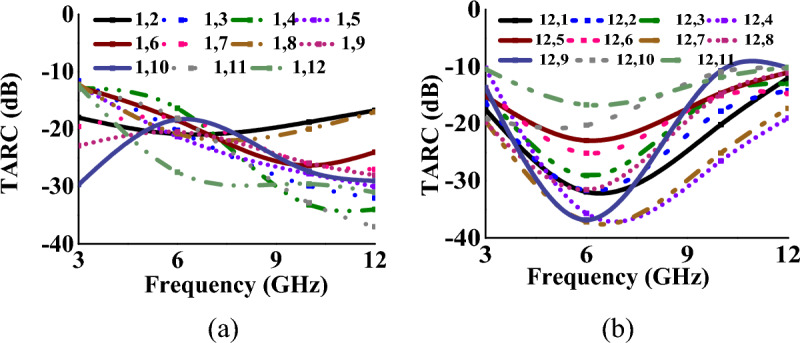
TARC characteristics at (w.r.t. other ports for Mode-I: wideband) (**a**) port-1, (**b**) port-12.

**Figure 15:**
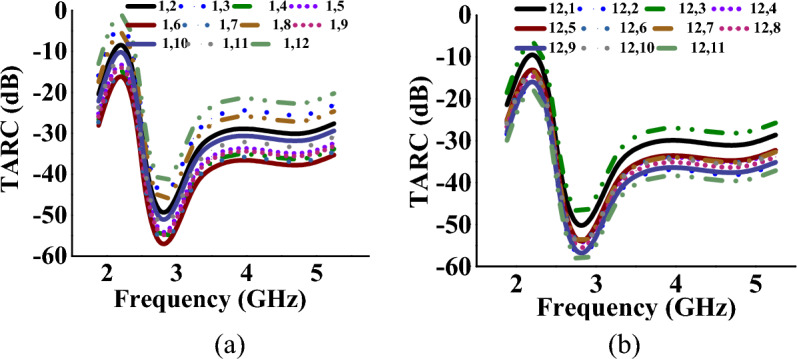
TARC characteristics at (w.r.t. other ports for Mode-II: multiband) (**a**) port-1, (**b**) port-12.

**Figure 16:**
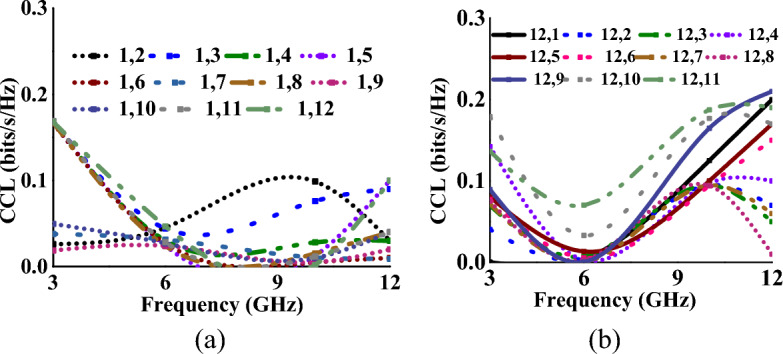
CCL characteristics at (w.r.t. other ports for mode-I: wideband) (**a**) port-1, (**b**) port-12.

**Figure 17:**
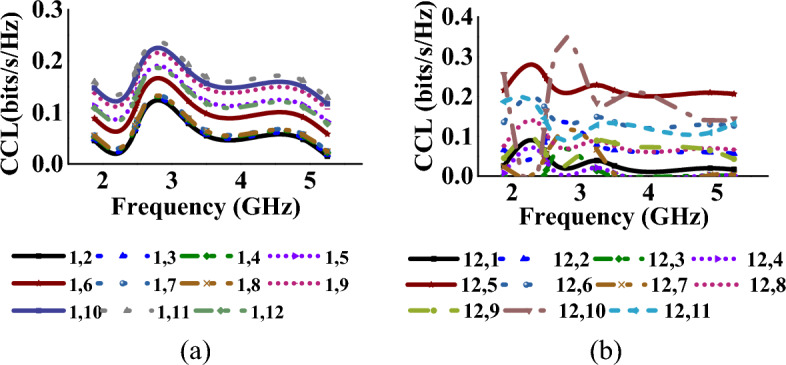
CCL characteristics at (w.r.t. other ports for mode-II: multiband) (**a**) port-1, (**b**) port-12.

TARC and CCL^32^ characterize the frequency, bandwidth, and radiation capability of multiport antennas and can be calculated using Eqs. (8) and (9)

8 $$\Gamma_{a}^{t} = \frac{{\sqrt {\sum\nolimits_{i = 1}^{N} {\left| {b_{i} } \right|^{2} } } }}{{\sqrt {\sum\nolimits_{i = 1}^{N} {\left| {a_{i} } \right|^{2} } } }}$$

9 $$CCL = - \log_{2} \det (\varphi^{R} )$$

where *φ*^*R*^ is the correlation matrix at the receiver side and *a*_*i*_ and *b*_*i*_ are incident and reflected signals, respectively. The TARC and CCL values (with respect to port-1, 5 and 12) of the MIMO antenna when operating in mode I and mode II, are represented in Tables 1, 2, 3, 4, 5, 6 respectively. In both cases, the antenna achieves TARC less than − 13 dB. The antenna CCL is less than 0.2 bits/s/Hz in mode I and less than 0.25 bits/s/Hz in mode II, which is less than the acceptable limit of 0.4 bits/s/Hz. Figures 14, 15, 16, 17 represent the TARC and CCL characteristics under different modes.

### Salient features of the proposed work

Table 7 compares the performance of the proposed MIMO antenna with the existing antenna designs. The proposed antenna is smaller in size, has more resonating bands, and offers frequency agility due to the use of PIN diodes and polarization and pattern diversity. The salient features of the proposed antenna configuration are:

- The antenna integrates two radiators in a small size of 26 mm × 26 mm, as compared to^10,19,20^, without any performance degradation.
- The antenna supports a wide range of applications, including UWB, GSM, Wi-Fi, LTE-7, 5G, Public safety WLAN, and WLAN than^10–20^ to provide high-speed communication without latency.
- The antenna also offers frequency agility, which helps to reduce interferences by turning ON and OFF the respective switches between UWB and multiple bands based on the user’s requirements, as compared to^10–12,14–16,18,19^.
- The MIMO set achieves greater than 14 dB isolation without the use of complex decoupling structures, and the twelve radiating elements are oriented in a 3-D fashion, with a size of 62 × 62 mm^2^ and inter-element spacing of 0.16 λ_0_.
- The proposed MIMO orientation helps in obtaining quad polarization, as compared to^10,12^, which suppress polarization mismatches in a highly scattering environment, thereby avoiding signal loss.
- The unique arrangements of antenna elements help in attaining multiple polarization vectors and pattern diversity with uncorrelated beams in both azimuthal and elevation planes.
- The antenna offers ECC < 0.08, TARC less than − 13 dB, and CCL < 0.25 bits/s/Hz, resulting in good diversity performance, as compared to^16,18,20^.
- The antenna's radiation efficiency is maintained at ~ 70 to 80% as the diodes are integrated with the transmission line without disturbing the radiator, reducing radiation losses.
- Thus, the proposed multiport antenna achieves spectrum efficiency and possesses better diversity characteristics to resolve connectivity issues in highly scattering environments.

**Table 7:** Performance comparison with existing antenna designs.

References	Antenna size (mm)	No. of operating bands	Polarization diversity	Isolation (dB)	Peak gain (dBi)	Efficiency (%)	Element spacing (*λ*_0_)	Reconfig-urability	No. of resonating elements
^10^	32 × 32	1**	Dual	> 15	4.2	60	–	No	2
^11^	38 × 90	1**	Dual	> 15	5	–	–	No	8
^12^	23 × 39.8	1**	Dual	> 20	5.1	82	–	No	2
^14^	70 × 55	1**	Dual	> 20	2.47	–	0.2	No	8
^15^	50 × 50	1**	Dual	> 17	3.15	–	–	No	8
^16^	40 × 40	3*	Dual	> 22	4.4	92	–	No	4
^18^	58 × 50	2*, 1**	Dual	> 20	0.2–0.6	–	–	No	8
^19^	100 × 100	2*, 1**	Hexa	> 20	2.1	90	0.24	No	12
^20^	90 × 80	3*, 1**	Tri	> 15	> 1.8	80–86	0.2	Yes	8
^33^	30 × 20	4*, 1**	Single	–	> 4.24	–	–	Yes	1
Prop	62 × 62	6*, 1**	Quad	> 15	5.2	70–80	0.16	Yes	12

*Narrowband, **Wideband.

## Conclusions

A multifunctional twelve-port polarization diversity antenna is presented with high link reliability, better connectivity, and a high data rate in ultra-dense scattering environments. The antenna offers a wide impedance bandwidth and stable radiation characteristics in both UWB and multiband modes. The antenna also offers multiple polarization vectors to avoid fading and cross-polarization. The diversity performance of the MIMO antenna is validated by measuring the ECC, TARC, and CCL. The proposed antenna could be useful for indoor wireless network communication scenarios such as smart buildings, smart factories, airports, and shopping malls to obtain high-speed communication.

## Data Availability

The datasets generated during and/or analysed during the current study are available from the corresponding author on reasonable request.
